# A Peculiar Case of Hybridism

**Published:** 1896-12

**Authors:** Cecil French

**Affiliations:** Washington, D. C.


### A PECULIAR CASE OF HYBRIDISM.

Reported by DR. CECIL FRENCH,
WASHINGTON, D. C.

Mr. Oyetton, student of the United States College of Veterinary Surgeons, brought here two specimens with the following history:

A mongrel cat lived and fraternized with a half-wild rabbit in a back-yard. In the course of time the cat, which was the female in the case, became possessed of two offspring. At six months of age they were about the size of a large kitten, apparently totally blind. Head showed predominance of cat, ears pink, teeth feline. They were very wild, would meow, and feces were like those of a cat. Hind-limbs, especially in the gait and tail, resembled a rabbit. Hair was rabbit-like. Hopped like a rabbit. Carnivorous in diet. There appeared to be some nervous affection, as they continually shook their heads from side to side.

---

The *Horseshoers' Journal*, in its new dress and under its new managerial direction, makes its appearance with the November number with the best of promises in fulfilling a stronger place of usefulness for the craft.

